# Barriers and enhancers to COVID-19 vaccination among healthcare workers in Zimbabwe

**DOI:** 10.4102/jphia.v16i1.719

**Published:** 2025-02-18

**Authors:** Nicholas Midzi, Clara Haruzivishe, Senga Sembuche, Masceline J. Mutsaka-Makuvaza, Rodgers Ayebare, Leah Mbabazi, Suzan Nakasendwa, Tonny Muwonge, Carl Mateta, Tafadzwa Madanhire, Cynthia Chaibva, Calleta Gwatiringa, Kudzaishe Mutsaka, Virginia Mawerewere, Isaac Phiri, Elizabeth Gonese, Tamrat Shaweno, Nebiyu Dereje, Raji Tajudeen, Mosoka Fallah, Munyaradzi Dobbie

**Affiliations:** 1 Health Systems Strengthening Research Unit, National Institute of Health Research, Ministry of Health and Child Care, Harare, Zimbabwe; 2 Department of Community Nursing, Faculty of Medicine and Health Science, University of Zimbabwe, Harare, Zimbabwe; 3 Africa Forum for Research and Education in Health, Harare, Zimbabwe; 4 Africa Centers for Disease Control and Prevention, Dar es Salaam, Tanzania; 5 Department of Microbiology and Parasitology, College of Medicine and Health, University of Rwanda, Kigali, Rwanda; 6 Infectious Diseases Institute, Makerere University, Kampala, Uganda; 7 National University of Science and Technology, Bulawayo, Zimbabwe; 8 Faculty of Medicine and Health Sciences, University of Zimbabwe, Harare, Zimbabwe; 9 Department of Epidemiology and Disease Control, Ministry of Health and Child Care, Harare, Zimbabwe; 10 Africa Centers for Disease Control and Prevention, Harare, Zimbabwe; 11 Africa Centers for Disease Control and Prevention, Addis Ababa, Ethiopia; 12 Africa Centers for Disease Control and Prevention, Monrovia, Liberia; 13 Public Health Division, Ministry of Health and Child Care, Harare, Zimbabwe

**Keywords:** SARS-CoV-2, COVID-19 vaccination, healthcare workers, barriers, enhancers

## Abstract

**Background:**

Coronavirus disease 2019 (COVID-19) vaccination is crucial for healthcare workers (HCWs). Understanding their vaccine uptake and perceptions is vital to promote acceptance.

**Aim:**

This study assessed COVID-19 vaccine uptake, associated factors and HCW willingness to recommend vaccination in Zimbabwe.

**Setting:**

The study was conducted through a cross-sectional survey involving 200 HCWs in seven central healthcare facilities from May 2023 to June 2023.

**Methods:**

Data on demographics, vaccination status, knowledge, attitudes and trust in information sources were collected. Descriptive statistics and modified Poisson regression identified factors associated with vaccine uptake.

**Results:**

Of the respondents (female: 68%, median age [interquartile range {IQR}: 34]; [28–43] years), 94% (188/200) had received at least one dose of the COVID-19 vaccine, with 49.5%, 41% and 3.5% having been fully vaccinated, received a booster and partially vaccinated, respectively. Also, 74% of the HCWs would recommend the COVID-19 vaccines to their patients. Out of the total, 15% of HCWs identified that vaccine safety was their major barrier to getting vaccinated. The vaccination rate among nurses and midwives was 9.6% (prevalence ratio [PR]: 0.904; 95% confidence interval [CI]: 0.833, 0.981) lower when compared to physicians. The study also identified that the booster vaccination rate was higher in older HCWs (PR: 1.02; 95% CI: 1.01, 1.03).

**Conclusion:**

High COVID-19 vaccine uptake was observed among HCWs in Zimbabwe. However, concerns about vaccine safety persist. Targeted interventions addressing these concerns are needed to maximise vaccine acceptance in this key population.

**Contribution:**

This study reveals specific reasons for vaccine hesitancy among HCWs in Zimbabwe.

## Introduction

The coronavirus disease 2019 (COVID-19) pandemic, caused by severe acute respiratory syndrome coronavirus 2 (SARS-CoV-2), has had a devastating impact worldwide. Coronavirus disease 2019 vaccines effectively reduce transmission, morbidity and mortality.^1^ Mass vaccination is one sure way to control the pandemic and safeguard lives and livelihoods on the continent. Mass vaccination is a proven strategy for protecting susceptible individuals in the population.^2^ Healthcare workers (HCWs) have played a vital role in the response to the virus.^3,4^ Vaccination against COVID-19 is crucial for protecting HCWs and ensuring the continuity of healthcare services.^5,6,7^ However, vaccine hesitancy and other barriers may hinder the uptake of COVID-19 vaccines among this high-risk population.^8,9^ A global survey examining why HCWs hesitate to receive vaccinations revealed several key factors. These included apprehensions about vaccine safety and side effects, the rapid pace of vaccine development and a general distrust in the processes behind vaccine mandates.^10,11^

At the start of vaccination campaigns in Zimbabwe, because of the scarcity of vaccines, priority was given to health workers, teachers and people of advanced age. However, the need for global vaccine equity led to improved supply of vaccines across Africa. As of late 2022, Africa has received up to 729 million doses of COVID-19 vaccines, and only 24.9% of those eligible had completed the primary vaccination series.^12^ In Zimbabwe, progress as of the same date showed that about 58.1% of individuals had received their initial vaccine dose, 43.5% had completed their vaccination course and 10.4% had obtained a booster dose, highlighting a mixed response in vaccine uptake within the region.^13,14^ Vaccines are easily accessible in the country, and the eligible population for vaccination was extended to include children from 12 years. Despite the increased access to efficacious COVID-19 vaccines, many remain unvaccinated because of prevailing misconceptions about the vaccines.^15^ This observation is often referred to as vaccine hesitancy. Vaccine hesitancy is a delay in the acceptance or refusal of vaccines despite the availability of vaccine services. It includes individuals who refuse or delay vaccinations or remain uncertain about the use of vaccines. The goal of effective management and prevention of COVID-19 in Zimbabwe significantly depends on the country’s ability to inoculate up to 70% of eligible persons by 2022.^2^ In this regard, we sought to understand barriers and enhancers to COVID-19 vaccine uptake among HCWs in Zimbabwe. This study distinguishes itself through a concentrated analysis of COVID-19 vaccine uptake among HCWs in Zimbabwe’s two largest cities, where access to vaccines is extensively guaranteed. We probed into the intricate cognitive and social dynamics shaping HCWs’ decisions regarding vaccination and their propensity to recommend COVID-19 vaccines to others with the aim of bolstering risk communication strategies and vaccine advocacy efforts.

## Research methods and design

### Study design and setting

We conducted a cross-sectional survey among HCWs at seven central health facilities from the Harare and Bulawayo metropolitan provinces of Zimbabwe between May 2023 and June 2023. These health facilities were the hotspots for the COVID-19 pandemic in the country.

### Study population and sampling strategy

The study population included physicians, nursing and midwifery personnel, and other HCWs like radiographers. The study purposively enrolled seven central hospitals from Bulawayo and Harare, from which we intentionally targeted a sample of 200 HCWs from different departments for participation at the facilities. The sample size of 200 was based on the Kish and Leslie formula, with a margin of error (precision) of 5.5. Participants were selected based on their profession and likelihood to interact with patients. The sample therefore included physicians, nurses and midwives, pharmacy personnel, laboratory personnel, community support and public health workers, and other allied health professionals (e.g. radiographers and anaesthetists).

### Data collection

We adopted a standardised structured questionnaire, which was administered to assess the HCWs’ uptake, willingness, attitudes and barriers towards COVID-19 vaccines based on the behavioural and social drivers model for vaccine demand creation. All data were entered into Case Report Forms (CRFs) hosted on the Research Electronic Data Capture (REDCap) software using mobile gadgets. The study variables included participant demographics; vaccination status; confidence in COVID-19 vaccines; reasons why HCWs would not want to get vaccinated; factors that would enable HCWs that have not been boosted or fully vaccinated to complete the vaccine schedule; preferences for the ideal place to get vaccinated and commonly used sources of information about vaccines and level of trust in the stated sources.

### Data analysis

Data were first exported from REDcap into STATA software version 17 for Windows 11 for management, checking data cleanliness, completeness and consistency of responses. Participant age was first assessed for normality using histograms and the Shapiro–Wilk test and presented as means (standard deviation) and medians (interquartile range [IQR]). All categorical variables (e.g. sex and age groups) are presented as frequencies and percentages, and comparisons were performed using the Chi-square test.

We first described the participants’ demographic and work-related characteristics by sex. We present the different COVID-19 vaccination status groups, comparing the differences in the participants’ demographic and work-related characteristics by vaccination status.

Descriptions in figures of the: (1) preferred centre to get a COVID-19 vaccine, (2) likelihood to recommend the vaccine to eligible individuals, (3) attitudes towards different vaccines, (4) ease of vaccination services, (5) barriers to being vaccinated, and (6) trust in information sources are presented in bar graphs and pie charts.

In addition, a modified Poisson regression model was used to identify the participants’ demographic and work-related characteristics associated with: (1) overall uptake of the COVID-19 vaccine among HCWs (at least one dose) and (2) uptake of the COVID-19 booster vaccine. After the bivariate analysis, factors with a *p*-value of ≤ 0.25 were included in the multivariable model.

The regression model prevalence ratios (PRs) and their 95% confidence interval (CI) exclude participants with unknown vaccination status.

### Ethical considerations

Ethical clearance to conduct this study was obtained from the Medical Research Council of Zimbabwe (reference no.: MRCZ/A/2987). Individual consent was sought from each participant, and written consent was obtained before each interview. De-identified data were entered into REDcap, and the hard copy questionnaires were kept under lock and key in a cabinet only accessible to the principal investigator.

## Results

### Participants’ characteristics

The study had 200 participants, primarily females (*n* = 136; 68.0%) with a median age of 34 (IQR: 28–43). Nurses and midwives (*n* = 72; 34.5%) accounted for a larger proportion of participants, followed by physicians (*n* = 49; 23%). The study identified that participants who were vaccinated were relatively older (median: 35, IQR: 28–44 years) as compared to those unvaccinated (median: 32, IQR: 25–39 years), although it was not statistically significant. Seventy per cent of those who highlighted not being vaccinated were from public hospitals (Table 1). However, it should be noted that the overall vaccine uptake by health facilities was uniform across different health facilities.

**TABLE 1:** Comparison of vaccinated and unvaccinated participants.

Variable	Category	Total (*n* = 198)				Unvaccinated (*n* = 10)				Vaccinated (*n* = 188)				*p*
		*n*	%	Median	IQR	*n*	%	Median	IQR	*n*	%	Median	IQR	
Age (years)	-	-	-	34.5	28–44	-	-	32	25–39	-	-	35	28–44	0.250
Age groups (years)	< 30	67	33.8	-	-	4	40.0	-	-	63	33.5	-	-	0.860
	30–39	69	34.8	-	-	4	40.0	-	-	65	34.6	-	-	-
	40–49	40	20.2	-	-	1	10.0	-	-	39	20.7	-	-	-
	50+	22	11.1	-	-	1	10.0	-	-	21	11.2	-	-	-
Sex	Female	136	68.7	-	-	5	50.0	-	-	131	69.7	-	-	0.190
	Male	62	31.3	-	-	5	50.0	-	-	57	30.3	-	-	-
Job title	Physicians	44	22.2	-	-	0	0.0	-	-	44	23.4	-	-	0.017
	Nursing and midwifery	69	34.8	-	-	5	50.0	-	-	64	34.0	-	-	-
	Pharmaceutical personnel	13	6.6	-	-	1	10.0	-	-	12	6.4	-	-	-
	Laboratory health workers	30	15.2	-	-	1	10.0	-	-	29	15.4	-	-	-
	Other health workers (e.g. radiographers and anaesthetists)	36	18.2	-	-	1	10.0	-	-	35	18.6	-	-	-
	Community support and public health workers	6	3.0	-	-	2	20.0	-	-	4	2.1	-	-	-
Health facility	Mater Dei	16	8.1	-	-	0	0.0	-	-	16	8.5	-	-	0.140
	Mpilo	24	12.1	-	-	1	10.0	-	-	23	12.2	-	-	-
	Parirenyatwa	61	30.8	-	-	5	50.0	-	-	56	29.8	-	-	-
	St Annes	23	11.6	-	-	3	30.0	-	-	20	10.6	-	-	-
	Sally Mugabe	37	18.7	-	-	0	0.0	-	-	37	19.7	-	-	-
	United Bulawayo	29	14.6	-	-	0	0.0	-	-	29	15.4	-	-	-
	Wilkins	8	4.0	-	-	1	10.0	-	-	7	3.7	-	-	-
Health facility sector	Public	158	79.8	-	-	7	70.0	-	-	151	80.3	-	-	0.430
	Private	40	20.2	-	-	3	30.0	-	-	37	19.7	-	-	-

IQR, interquartile range.

#### Uptake of COVID-19 vaccines

The majority (*n* = 188; 94%) of the HCWs had received at least one dose. Specifically, half (*n* = 101; 50.5%), two in five (*n* = 82; 41%) and seven (3.5%) of the participants reported to have been fully vaccinated, received a booster dose and were partially vaccinated, respectively.

**FIGURE 1:**
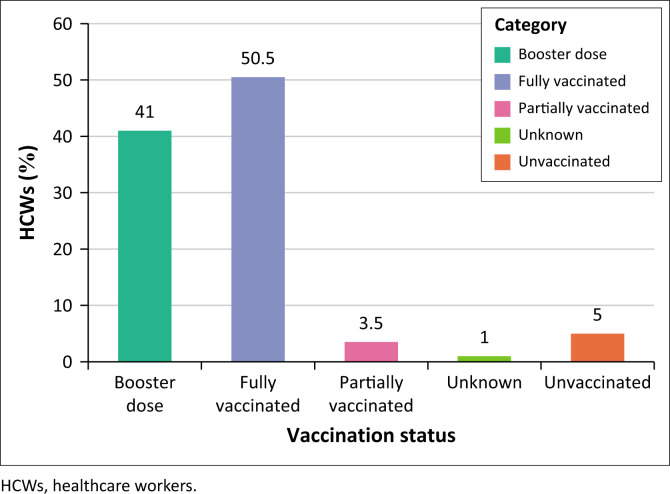
Vaccination status of healthcare workers.

However, we also report that 5% (*n* = 10) of the HCWs were not vaccinated for COVID-19, while two participants refused to disclose their vaccination status (Figure 1).

#### Preference centres to receive COVID-19 vaccine doses

**FIGURE 2:**
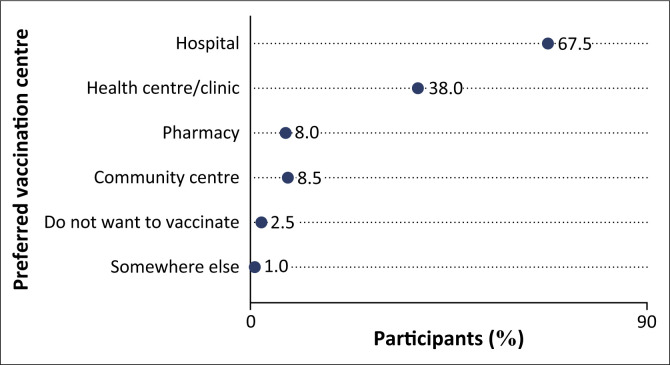
Preference of vaccination centres among healthcare workers.

The responses from HCWs showed that the majority (67.5%) preferred receiving the vaccine at hospitals, while just over a third (38%) opted for other small health centres or clinics. In addition, 8% (*n* = 16) of the participants indicated pharmacies as a preferred choice to get a vaccine dose, with an almost equal proportion choosing community centres (8.5%). Interestingly, 2.5% (*n* = 5) of the participants did not want anything to do with COVID-19 vaccines (Figure 2).

### Likelihood to recommend COVID-19 vaccination to eligible individuals

**FIGURE 3:**
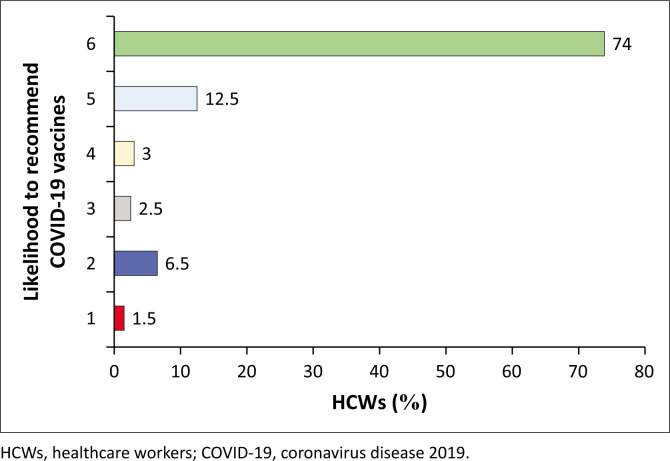
Likelihood to recommend COVID-19 vaccination to eligible individuals.

The study reports that the majority (*n* = 173; 86.5%) of the participants indicated that they would either definitely (*n* = 148; 74%) or probably (*n* = 25; 12.5%) likely to recommend the COVID-19 vaccines to eligible individuals in communities. However, a sizeable number (*n* = 27; 13.5%) reported that they would probably (*n* = 6; 3%) and definitely (*n* = 5; 2.5%) not recommend respectively, while 6.5% (*n* = 13) were unsure and 1.5% (*n* = 3) did not wish to respond to the question (Figure 3).

### Recommendation of available COVID-19 boosters to eligible individuals

**FIGURE 4:**
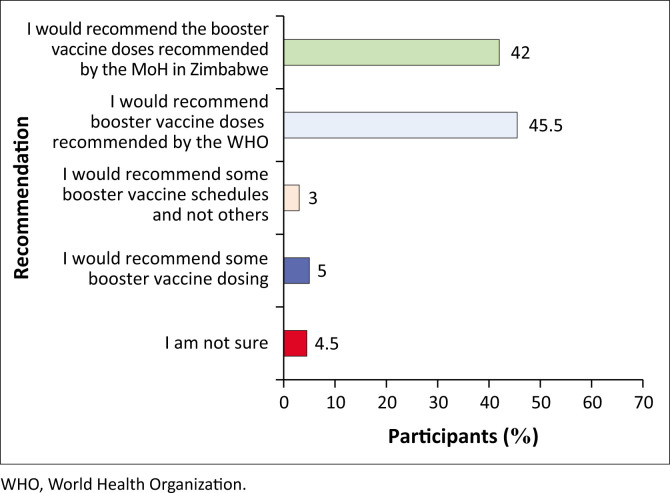
Recommendation of available COVID-19 boosters to eligible individuals.

Most HCWs indicated they would recommend vaccine doses recommended by either the World Health Organization (WHO) (*n* = 90; 45.5%) or the health authorities in Zimbabwe (*n* = 84; 42%). The HCWs also mentioned that they would recommend some booster doses (*n* = 16; 8%), although nine (4.5%) participants also pointed out that they were not sure (Figure 4).

### Level of confidence in vaccines against diseases

**FIGURE 5:**
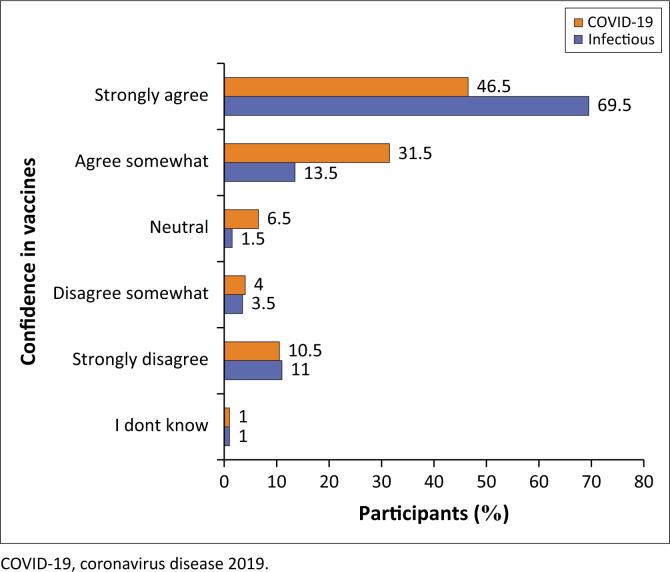
Level of confidence in vaccines against COVID-19 and other infectious diseases.

In this study, most HCWs strongly agree that vaccines work against COVID-19 (46.5%) and other infectious diseases (69.5%). More specifically, it should be noted that the strength of belief in how vaccines work was lower for COVID-19 than for other infectious diseases. As such, more HCWs agreed somewhat (*n* = 63; 31.5%) that COVID-19 vaccines work, while only 27 (13.5%) pointed to the same for other infectious diseases. One-tenth of the participants strongly disagreed that vaccines work for both COVID-19 (*n* = 21; 10.5%) and other infectious diseases (*n* = 22; 11%) (Figure 5).

### Accessibility of vaccination services

**FIGURE 6:**
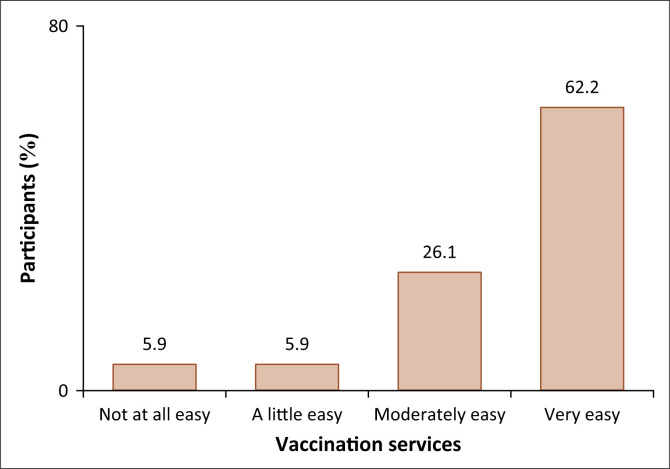
Ease of vaccination services.

Almost two-thirds of the participants (*n* = 117; 62.2%) mentioned that accessing vaccination services is very easy, while a quarter (*n* = 49; 26.1%) found it moderately easy. In addition, 11 of the enrolled HCWs mentioned that it is a little easy, and another 11 found it not easy at all (Figure 6).

#### Confidence in answering patient questions about COVID-19

**FIGURE 7:**
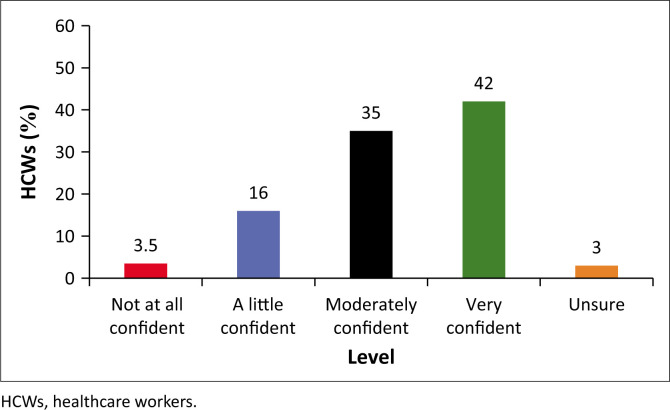
Level of confidence in answering patient questions about COVID-19.

The HCWs were also asked about their confidence in responding to patient questions about COVID-19. Notably, 93.5% indicated they were overall confident on different levels (a little: 16%; moderately: 35% and very confident: 42%). However, 6.5% (*n* = 13) of the participants were either unsure (3%) or not at all confident (3.5%) to respond to patient questions about COVID-19 (Figure 7).

#### Barriers to COVID-19 vaccination or booster doses

**FIGURE 8:**
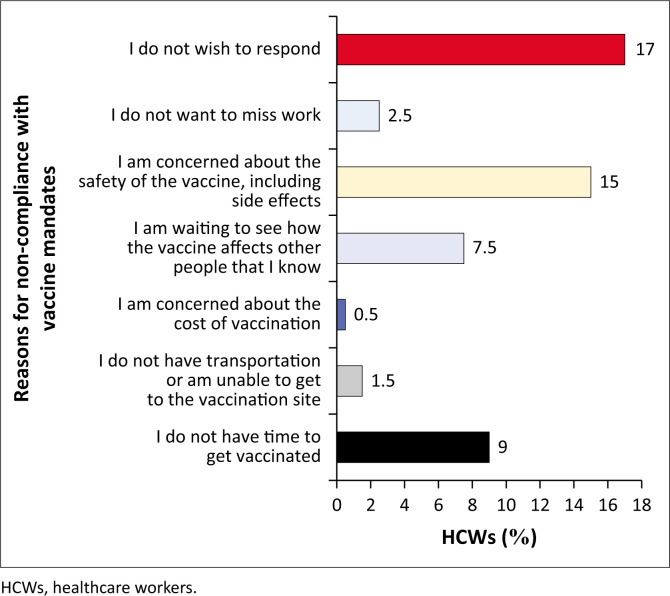
Reasons why the healthcare workers are not yet fully vaccinated and/or boosted.

Among those who were partially vaccinated (not received a complete regimen of doses) and/or not yet received a booster dose, 15% of the participants mentioned vaccine safety as the primary barrier and waiting to see the vaccine effect on other people (7.5%) and a lack of time to get all doses (9%) as the main reasons for not being vaccinated (Figure 8).

**FIGURE 9:**
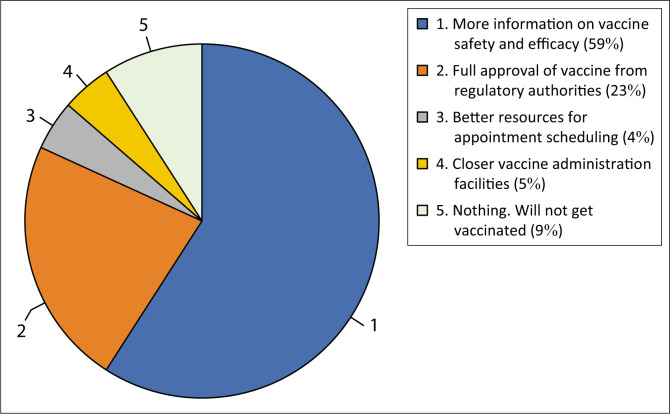
Factors that would help healthcare workers in the decision to get vaccinated or boosted.

When asked what should be improved to help them decide to get vaccinated or boosted, more than half (59%) of the HCWs mentioned that they need more information on vaccine safety and efficacy. In comparison, almost a quarter (23%) required the full approval of vaccines from regulatory authorities (Figure 9). On the other hand, 4% and 5% of the participants pointed out better resources for appointment rescheduling and closer vaccine administration facilities, respectively.

#### The level of trust in different information sources among healthcare workers

Half of the respondents (*n* = 100; 50%) and more than a third (*n* = 77; 38.5%) either entirely or mainly trust the information given by the Zimbabwe Ministry of Health authorities, respectively. In addition, almost a quarter of the participants acknowledged their complete trust in local (*n* = 47; 23.5%) and international television channels (*n* = 49; 24.5%).

Three in five HCWs (*n* = 119; 59.5%) also mentioned that they mainly trust their co-workers regarding COVID-19. Participants also mentioned that they mainly trust their bosses (*n* = 77; 38.5%), newspapers (*n* = 65; 32.5%), websites (*n* = 64; 32%) and local radio channels (*n* = 65; 32.5%).

**FIGURE 10:**
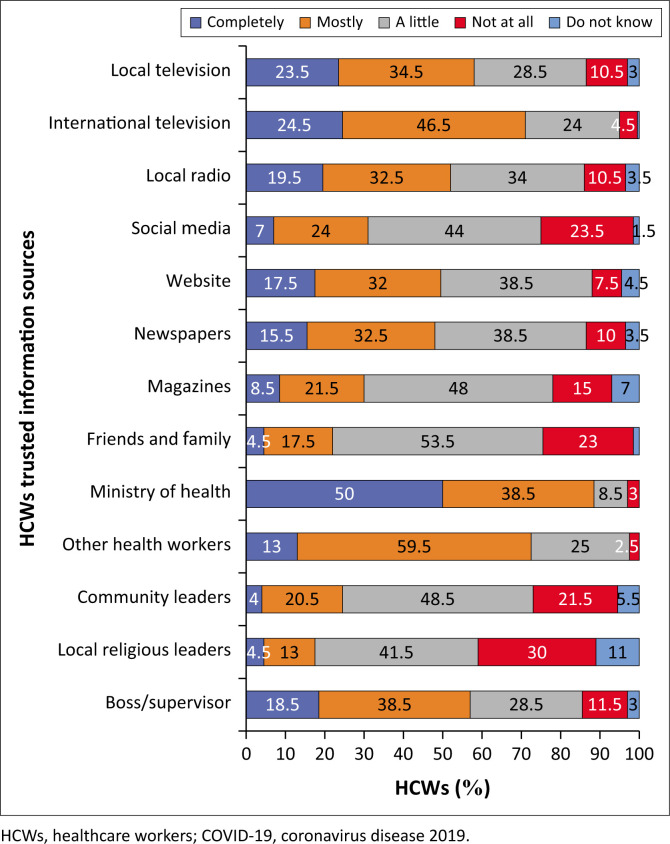
Trust in information sources about COVID-19 and vaccination.

More than half (*n* = 107; 53.5%) and 44% of the HCWs also said they somewhat trust their family, friends and social media. However, the most untrusted sources of information among the participants were local religious leaders (30%), social media (23.5%), friends and family (23%) and community leaders (21.5%) (Figure 10).

#### Factors associated with overall uptake of COVID-19 vaccines

Notably, we report that the adjusted vaccination rate in nurses and midwives was 9.6% (adjusted PR: 0.904; 95% CI: 0.833–0.981) lower when compared to physicians.

We also identified that the unadjusted vaccination rate in Harare was 5.6% (PR: 0.944; 95% CI: 0.893–0.998) compared to Bulawayo, although the effect was attenuated in the multivariable analysis. On the other hand, age, sex and job title were not associated with vaccine uptake among HCWs in this study (Table 2).

**TABLE 2:** Regression analysis for the association of demographic characteristics and uptake of COVID-19 vaccine.

Variable	Category	Unadjusted PR		Adjusted PR	
		Median	IQR	Median	IQR
Age (years)	-	1.002	0.998, 1.005	-	
Age groups (years)	< 30	Ref	Ref	Ref	Ref
	30–39	1.002	0.921, 1.090	1.016	0.941, 1.100
	40–49	1.037	0.959, 1.121	1.058	0.975, 1.148
	50+	1.015	0.910, 1.133	1.044	0.954, 1.142
Sex	Female	Ref	Ref	Ref	Ref
	Male	0.954	0.880, 1.035	0.947	0.863, 1.040
Job title	Physicians	Ref	Ref	Ref	Ref
	Nursing and midwifery	0.927**	0.868, 0.991**	0.904	0.833, 0.981**
	Pharmaceutical personnel	0.923	0.789, 1.080	0.939	0.795, 1.108
	Laboratory health workers	0.967	0.904, 1.033	0.961	0.890, 1.037
	Other health workers (e.g. radiographers and anaesthetists)	0.972	0.920, 1.027	0.958	0.896, 1.024
	Community support and public health workers	0.667	0.378, 1.175	0.678	0.381, 1.203
Health facility site	Bulawayo	Ref	Ref	Ref	Ref
	Harare	0.944**	0.893, 0.998**	0.960	0.912, 1.011
Health facility sector	Public	Ref	Ref	Ref	Ref
	Private	0.968	0.880, 1.064	0.998	0.911, 1.092

PR, prevalence ratio; IQR, interquartile range; COVID-19, coronavirus disease 2019; Ref, reference.

** , *p* < 0.05.

#### Factors associated with overall uptake of COVID-19 booster vaccines

The multivariable Poisson regression analysis for the exposures of COVID-19 booster vaccine uptake showed that the booster vaccination rates increased by 2% (adjusted PR: 1.019, 95% CI: 1.004–1.034) for every 1-year age increase among HCWs. Conversely, the overall adjusted model showed that COVID-19 booster vaccination rates were 33.3% (adjusted PR: 0.667, 95% CI: 0.486–0.914) lower in Harare than in Bulawayo. Furthermore, compared to physicians, COVID-19 booster vaccination rates among community support and public health workers were at least 90% lower (Table 3).

**TABLE 3:** Regression analysis for the association of demographic characteristics and uptake of a booster COVID-19 vaccine.

Variable	Category	Unadjusted PR		Adjusted PR	
		Median	IQR	Median	IQR
Age (years)	-	1.020	1.005, 1.036**	1.019	1.004, 1.034*
Age groups (years)	< 30	Ref	Ref	Ref	Ref
	30–39	2.124	1.305, 3.460*	2.195	1.355, 3.556*
	40–49	1.989	1.160, 3.409**	2.230	1.329, 3.741*
	50+	1.903	1.016, 3.566**	1.857	1.011, 3.411**
Sex	Female	Ref	Ref	Ref	Ref
	Male	0.997	0.691, 1.438	1.056	0.732, 1.524
Job title	Physicians	Ref	Ref	Ref	Ref
	Nursing and midwifery	1.052	0.700, 1.582	0.916	0.590, 1.422
	Pharmaceutical personnel	0.846	0.395, 1.812	1.010	0.474, 2.152
	Laboratory health workers	0.660	0.349, 1.248	0.553	0.306, 1.001
	Other health workers (e.g. radiographers and anaesthetists)	0.794	0.461, 1.378	0.719	0.436, 1.185
	Community support and public health workers	0.000000817	0.000000344, 0.00000194*	0.000000617	0.000000240, 0.000000158*
Health facility site	Bulawayo	Ref	Ref	Ref	Ref
	Harare	0.622	0.447, 0.864*	0.667	0.486, 0.914**
Health facility sector	Public	Ref	Ref	Ref	Ref
	Private	0.911	0.585, 1.419	0.846	0.548, 1.307

PR, prevalence ratios; IQR, interquartile range; COVID-19, coronavirus disease 2019; Ref, reference.

* , *p* < 0.001;

** , *p* < 0.05.

## Discussion

This study examined the level of uptake of the COVID-19 vaccine and identified barriers and enhancers to COVID-19 vaccination among HCWs in selected central hospitals in Zimbabwe. We found that 94% of HCWs had received at least one vaccine dose, with 50.5% fully vaccinated. Age and occupation were associated with vaccination status, with older HCWs and physicians more likely to be vaccinated, and this observation concurs with other studies, including one by Weinerman et al., 2023.^16,17^ Nurses had lower vaccination rates than physicians, and age influenced booster vaccine uptake. The observation that older HCWs were more likely to be vaccinated than younger ones was contrary to the findings from a general population study in Zimbabwe.^17^ However, there was concurrence with this study on the trend that there is more vaccine hesitancy in women than in men.^17^ Although the reasons for this disparity were not investigated, this could be linked to considerations of the fear that vaccination poses a risk of reduced fertility in women of childbearing age that circulated in unconfirmed social media reports. While older age is often associated with higher vaccination rates because of the increased risk of severe illness, as Nzaji et al., 2024^18^ identified in Congo, variations in access to healthcare, vaccine hesitancy and occupational factors may influence vaccination uptake among HCWs.^19^ Additionally, differences in vaccine distribution strategies, messaging campaigns and perceptions of vaccine safety and efficacy within the HCW population could contribute to divergent patterns compared to the general population.^20^

Most HCWs recommended COVID-19 vaccination to eligible individuals and preferred receiving vaccines at hospitals. As reported by Agyekum et al., 2021 in Ghana,^21^ the high recommendation rate for COVID-19 vaccination among HCWs reflects their role as trusted sources of medical advice, potentially influencing public vaccine acceptance. Their preference for vaccination at hospitals may stem from familiarity with healthcare settings, perceived convenience and confidence in the vaccine administration and monitoring infrastructure. Trust was placed in the Zimbabwe Ministry of Health and Child Care and co-workers for information on COVID-19. Despite having half of the HCWs fully vaccinated, most were willing to recommend the COVID-19 vaccines to eligible persons. Similarly, a study by Lubad et al. in Jordan revealed that most health workers were willing to recommend vaccination for other people, mainly high-risk clients.^22^

Vaccine hesitancy was high among HCWs, thus causing a delay in their being fully vaccinated. It is also noted that participants had lower confidence in COVID-19 vaccines than other established ones because of safety concerns about COVID-19 vaccines. Coronavirus disease 2019 vaccine hesitancy was high among health workers in Tanzania (46.6%) and Zambia (27.9%).^20,21^ This finding was attributed to safety concerns exacerbated by misinformation and inadequate knowledge about the vaccine and its efficacy. A marginal decrease in confidence about the uptake of COVID-19 vaccines among health workers was noted compared to other non-COVID-19 vaccines, possibly because of fears of vaccine safety and efficacy. This finding could explain the delay in initiating or completing their vaccination schedules. These findings are supported by a study on HCWs in Zambia that revealed a low uptake rate because of concerns regarding suspected side effects, doubt about the efficacy, and a lack of confidence and trust in the vaccines themselves.^23^

A key limitation of this study was that the sample was limited to two metropolitan provinces of Zimbabwe. Although these had the highest cases of COVID-19, the sample did not consider the rural sample. Furthermore, a limitation of this study is its relatively small sample size, which may restrict the generalisability of our quantitative findings to the broader population. Although the gender distribution observed is generally what is obtained in the workforce, the fact that the majority of participants in the study were women may have influenced the outcome given that they are more likely to have elevated safety concerns. Caution should be exercised in extending the results beyond the study sample, as it may not fully represent the wider demographic characteristics and variations.

## Conclusion

The results of this study have shown a high uptake of the initial COVID-19 dose among HCWs (94%), with half of them (50.5%) having been fully vaccinated. Most HCWs recommended COVID-19 vaccination to eligible individuals (86.5%) and preferred to receive vaccines at hospitals (67.5%). Vaccine safety and a lack of information were identified as barriers to vaccination, with resultant hesitancy and uncertainty about vaccine efficacy. The uptake of the COVID-19 vaccination was affected by inadequate, misleading and inaccurate information regarding safety and efficacy issues. This meant HCWs could not rely on one information source but had to triangulate information from various sources to make decisions and to be able to advise patients and those around them. These findings contribute to the global efforts to combat the COVID-19 pandemic by ensuring the protection of HCWs and enhancing overall vaccination coverage in Africa. Although this article addresses COVID-19 vaccines, which were relevant during the pandemic, the results are important as they reflect healthcare workers’ attitudes towards newly developed vaccines. This information will be useful in future pandemics.
